# Development and validation of nomograms for predicting the risk probability of carbapenem resistance and 28-day all-cause mortality in gram-negative bacteremia among patients with hematological diseases

**DOI:** 10.3389/fcimb.2022.969117

**Published:** 2023-01-05

**Authors:** Xing Jian, Shuaixian Du, Xi Zhou, Ziwei Xu, Kejing Wang, Xin Dong, Junbin Hu, Huafang Wang

**Affiliations:** ^1^ Institute of Hematology, Union Hospital, Tongji Medical College, Huazhong University of Science and Technology, Wuhan, China; ^2^ Department of Clinical Laboratory, Union Hospital, Tongji Medical College, Huazhong University of Science and Technology, Wuhan, China

**Keywords:** gram-negative bacteria, carbapenem resistance, mortality, prediction, nomogram, bloodstream infections

## Abstract

**Objectives:**

Gram-negative bacteria (GNB) bloodstream infections (BSIs) are the most widespread and serious complications in hospitalized patients with hematological diseases. The emergence and prevalence of carbapenem-resistant (CR) pathogens has developed into a considerable challenge in clinical practice. Currently, nomograms have been extensively applied in the field of medicine to facilitate clinical diagnosis and treatment. The purpose of this study was to explore risk indicators predicting mortality and carbapenem resistance in hematological (HM) patients with GNB BSI and to construct two nomograms to achieve personalized prediction.

**Methods:**

A single-center retrospective case-control study enrolled 244 hospitalized HM patients with GNB-BSI from January 2015 to December 2019. The least absolute shrinkage and selection operator (LASSO) regression analysis and multivariate logistic regression analysis were conducted to select potential characteristic predictors of plotting nomograms. Subsequently, to evaluate the prediction performance of the models, the prediction models were internally validated using the bootstrap approach (resampling = 1000) and 10-fold cross validation.

**Results:**

Of all 244 eligible patients with BSI attributed to GNB in this study, 77 (31.6%) were resistant to carbapenems. The rate of carbapenem resistance exhibited a growing tendency year by year, from 20.4% in 2015 to 42.6% in 2019 (*p* = 0.004). The carbapenem resistance nomogram constructed with the parameters of hypoproteinemia, duration of neutropenia ≥ 6 days, previous exposure to carbapenems, and previous exposure to cephalosporin/*β*-lactamase inhibitors indicated a favorable discrimination ability with a modified concordance index (C-index) of 0.788 and 0.781 in both the bootstrapping and 10-fold cross validation procedures. The 28-day all-cause mortality was 28.3% (68/240). The prognosis nomogram plotted with the variables of hypoproteinemia, septic shock, isolation of CR-GNB, and the incomplete remission status of underlying diseases showed a superior discriminative ability of poorer clinical prognosis. The modified C-index of the prognosis nomogram was 0.873 with bootstrapping and 0.887 with 10-fold cross validation. The decision curve analysis (DCA) for two nomogram models both demonstrated better clinical practicality.

**Conclusions:**

For clinicians, nomogram models were effective individualized risk prediction tools to facilitate the early identification of HM patients with GNB BSI at high risk of mortality and carbapenem resistance.

## 1 Introduction

Bloodstream infections (BSIs) are the most widespread and serious infectious complications in hospitalized patients with hematological diseases, characterized by significant high morbidity, mortality, and a heavy additional medical burden, primarily arising from the underlying diseases themselves and the cytotoxic chemotherapy-related immunosuppression (Feld, 2008; Misch and Andes, 2019). During recent years, there has been a pronounced trend reversal as regards the bacterial spectrum of bacteremia in patients with neutropenia and hematologic malignancies, with Gram-negative bacteria (GNB) being the most commonly reported pathogenic organisms nowadays (Trecarichi and Tumbarello, 2014; Trecarichi et al., 2015; Chen et al., 2021). In addition, owing to the high selection pressure of antimicrobial agents, a considerable increase has been observed in the infection of multiple antibiotic-resistant strains that are insusceptible to a wide variety of antibiotics. In this context, the progressively emergence and rapidly spread of multidrug-resistant bacteria has been emerging as a globally major challenge, particularly for carbapenem-resistant (CR) GNB (Lalaoui et al., 2020). Considering that it normally takes 2 to 3 days to obtain the results of blood cultures and antimicrobial susceptibility tests against the background of current clinical laboratory conditions, inappropriate empirical antibiotic therapy is life-threatening for patients infected with these strains. Therefore, early identification of risk factors associated with carbapenem resistance and exploration of prognosis-related indicators is essential to purposefully improving clinical outcomes for hematological (HM) patients infected with GNB BSI.

Currently, nomograms have been diffusely applied in medical research as an effective complementary tool to implement clinical decisions for clinicians (Balachandran et al., 2015; Wu et al., 2020; Lin et al., 2020; Song et al., 2021; Dong et al., 2021; He et al., 2022). As a graphical tool, compared with traditional predictive scoring systems based on regression analysis, the nomogram can provide a visual representation of the complex statistical model and accurately estimate the individual probability of a clinical event by integrating multiple predictive variables that can diagnose diseases or predict clinical outcomes (Park, 2018; Jiang et al., 2022). However, nomograms predicting the risk factors of mortality and carbapenem resistance in HM patients with GNB BSI have rarely received attention. Consequently, the primary objective of this study was to develop and validate two clinical predictive models for the early individualized and accurate prediction of carbapenem resistance risk and 28-day all-cause mortality in HM patients suffering from GNB BSI, respectively.

## 2 Methods

### 2.1 Study setting and patient population

The retrospective case-control study was performed between January 2015 and December 2019 at the Institute of Hematology, Union Hospital, Tongji Medical College, Huazhong University of Science and Technology, a 5000-bed tertiary care teaching hospital in central China. Episodes of BSI were identified from the clinical microbiology laboratory database in accordance with the US Centers for Disease Control and Prevention (CDC) criteria (Horan et al., 2008). The first episodes of BSI caused by GNB that occurred in hospitalized hematological patients older than 16 years were included in this study. Meanwhile, patients who had been treated with hematopoietic stem cell transplantation before BSI were excluded, as well as those with polymicrobial bacteremia. Ultimately, 244 patients with GNB BSI met the inclusion and exclusion criteria were enrolled in the retrospective cohort.

### 2.2 Data collection and study design

**Figure 1:**
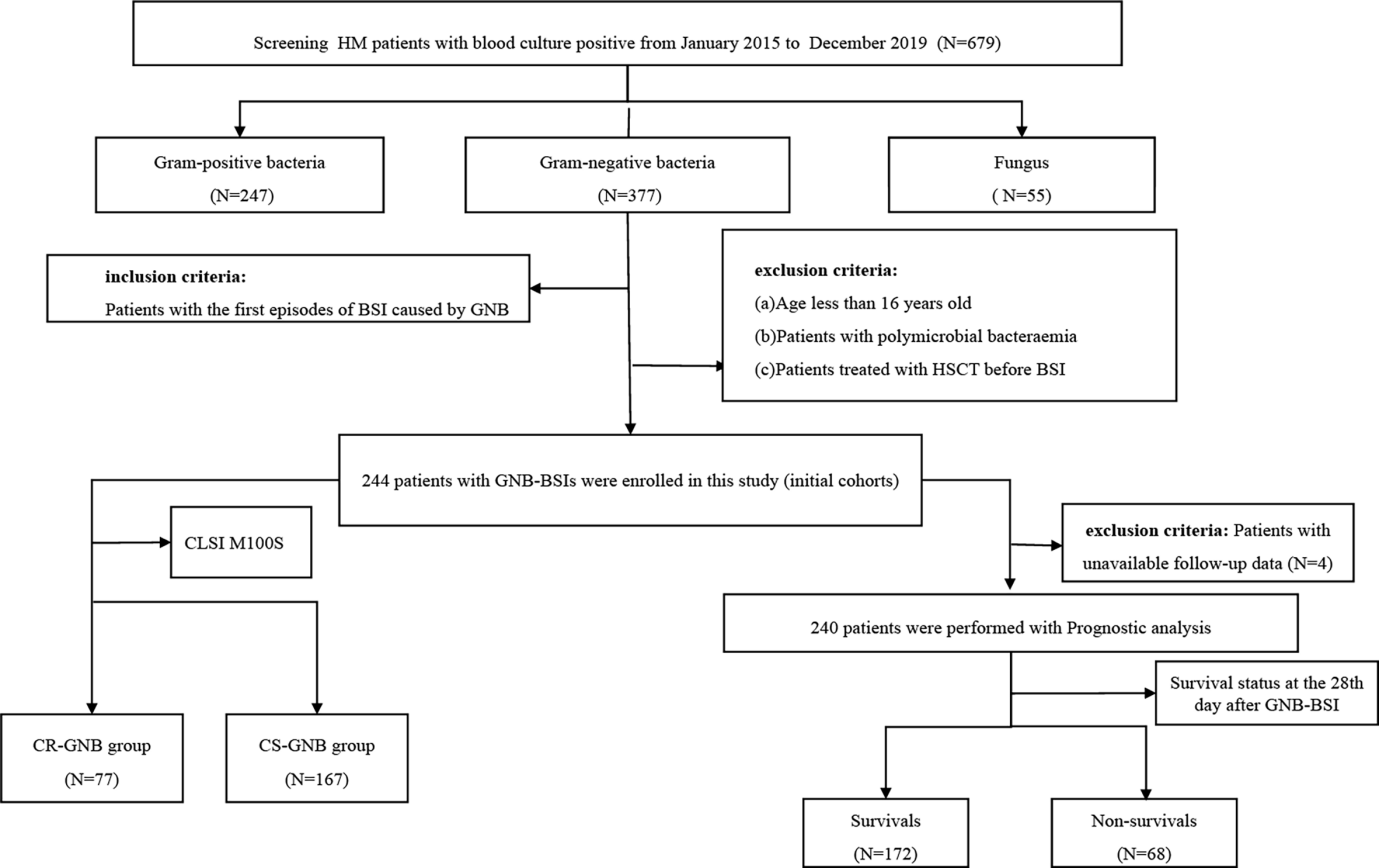
Flowchart of the study design. HM, hematological; CLSI,clinical and laboratory standards institute; GNB, gram-negative bacteria; BSI, bloodstream infection; CR, carbapenem-resistant; CS, carbapenem-sensitive.

Date extracted from the hospital microbiology laboratory database and the medical electronic medical record systems included demographics (age and sex), microbiological data, underlying diseases and the stage of disease at the time of GNB BSI, comorbidities (diabetes mellitus, cardiovascular disease, hepatic disease, pulmonary infection at the time of GNB BSI), the use of immunosuppressants and corticosteroids, invasive procedures and/or devices, antibiotic therapy, the presence and duration of neutropenia, clinical outcomes, and so on. The flowchart of study design was shown in **Figure 1**. For the purposes of developing a predictive model for carbapenem-resistant bacteremia in this study, patients were divided into the carbapenem-resistant (CR-GNB) groups (77 cases) and the carbapenem-sensitive (CS-GNB) groups (167 cases) based on the reports of antimicrobial susceptibility testing (AST). Simultaneously, we also analyzed the potential risk factors affecting mortality within 28 days following the onset of GNB BSI.

### 2.3 Definition

Hospital-acquired BSI was considered as the isolation of pathogens from at least one blood culture specimen after 48 hours of admission to hospital. The onset of BSI was defined as the collection date of a positive blood culture sample. Neutropenia was defined as an absolute neutrophil count (ANC) < 500 neutrophils/μl at the time of BSI onset. Corticosteroid therapy was defined as the administration of prednisone (dose ≥ 20mg/day, duration ≥ 5 days) or its equivalent. Immunosuppressive therapy referred to the use of at least one of cyclosporine, tacrolimus, rituximab, and ATG/ALG. Hypoproteinemia was defined as a serum albumin value of less than 30 g/L on the day (or within 24 hours) of the collection of a positive blood culture specimen. Mucosal barrier damage was defined as gastrointestinal mucositis or oropharyngeal mucositis. Antimicrobial agent exposure was defined as the use of antibiotics for more than 72 hours within 30 days before BSI. Typical antimicrobial agents administered within 30 days prior to BSI in this study included carbapenems (imipenem and meropenem), aminoglycosides (etimicin, amikacin and kanamycin), quinolones (levofloxacin and moxifloxacin), tigecyclines, penicillins/β-lactamase inhibitor combinations (piperacillin-tazobactam) and cephalosporins/β-lactamase inhibitor combinations (cefoperazone-tazobactam, cefoperazone-sulbactam, ceftazidime-tazobactam and ceftriaxone-tazobactam). The diagnosis of septic shock followed the Third International Consensus Definitions for Sepsis and Septic Shock (Sepsis-3) clinical criteria (Singer et al., 2016). Carbapenem resistance was defined as resistance to one or more of meropenem, imipenem, and ertapenem. Empirical antimicrobial therapy was considered appropriate when receiving at least one antimicrobial agent with *in vitro* activity within 48 hours after the episode of BSI. The final clinical outcome was determined by the survival status on the 28th day after the onset of GNB BSI. If a patient was discharged from a hospital within 28 days of the onset of BSI, the clinical outcome was determined by telephone follow-up.

### 2.4 Microbiological method

Microbial identification and antibiotic susceptibility testing were conducted in the clinical microbiology laboratory of hospital using a fully automated microbiology system (BD phoenix™-100). Antibiotic susceptibilities were interpreted according to the guidelines of the Clinical and Laboratory Standards Institute M100S (CLSI M100S), except for colistin and tigecycline, which were determined in accordance with the European Committee on Antimicrobial Susceptibility Testing (EUCAST) clinical breakpoints.

### 2.5 Derivation and performance evaluation of the nomogram

The least absolute shrinkage and selection operator (LASSO) regression model with ten-fold cross validation could avoid the multicollinearity of variables and minimize the possibility of model overfitting or underfitting (Iasonos et al., 2008; Friedman et al., 2010; Collins et al., 2015). Given the large number of predictors, the LASSO regression analysis and multivariate logistic regression analysis were performed to select potential characteristic predictors. Afterward, the clinical nomogram were formulated based on the potential characteristic predictors screened above and the significant predictors that had an essential impact on clinical outcomes. In the nomogram, each predictor was visually assigned a corresponding score. The accumulated total points for a clinical event could be calculated by adding the scores of each predictor, corresponding to the predicted probability of the clinical event.

The prediction performance of the nomogram model was evaluated with internal validation, which were primarily conducted using the bootstrap method (resampling = 1000) and 10-fold cross validation. In addition, to minimize the risk of bias of single-center studies, we further evaluate the stability of the prediction model using other cross validation methods such as hold-out cross validation (7:3), leave-one-out cross validation, and bootstrapping with 10-fold cross validation (resampling = 1000). The Concordance index (C-index) or area under the receiver operating characteristic (ROC) curve (AUC) was used to assess the discrimination ability of a predictive model. The C-index of a nomogram was equal to the AUC values of a logistic regression model since the ROC for the logistic regression model was drawn based on the predicted probability. Ordinarily, the C-index and AUC values > 0.7 are considered to have relatively good discriminative accuracy. The calibration curves were used to evaluate the calibration ability of a predictive model-that is, the ability of the predicted probabilities of clinical outcomes to be consistent with the actual probabilities. Besides this, decision curve analysis (DCA) was also performed to estimate the clinical application value of the model.

### 2.6 Statistical analysis

Data analysis was performed using SPSS 25.0 statistical software (SPSS, Chicago, IL, USA) and R software version 4.1.3. The R packages used for statistical analysis in the R project (version 4.1.3) mainly consisted of glmnet, rms, caret, pROC, and rmda. All statistical analyses were two-tailed, with *P* -values < 0.05 considered statistically significant. The Kolmogorov-Smirnov normality test was utilized to assess the normality distribution of continuous variables. Continuous variables were expressed as median (M) or interquartile range (IQR) and compared using the Mann-Whitney *U* test. Categorical variables were represented as numbers or percentages (%) of cases and analyzed using the Chi-squared test or the Fisher’s exact test. Survival curves were plotted based on the Kaplan-Meier method and compared using the log-rank test.

## 3 Results

### 3.1 Distributions of GNB isolates in the original study cohorts

**Figure 2:**
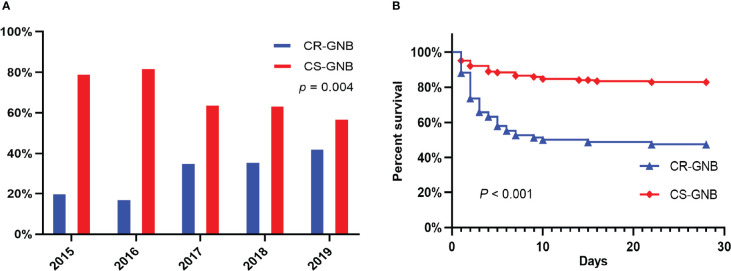
Percentages of Resistance to Carbapenems among Gram-negative bacteria during the Study Period **(A)**; Kaplan-Meier Survival Analysis among Hematological Patients with BSI caused by Carbapenem resistant (CR) and carbapenem susceptible (CS) Gram-negative bacteria **(B)**.

According to the inclusion and exclusion criteria, a total of 244 patients with GNB BSI were included in the study from January 2015 to December 2019. Of all the 244 isolates of GNB, 173 (70.9%) were of *Enterobacteriaceae*, 62 (25.4%) were *glucose non-fermenting* (GNF) GNB, and 9 (3.7%) were of *Vibrionaceae*. *Klebsiella pneumoniae* (KP) (n = 79) and *Escherichia coli* (EC) (n = 66) were the main members of the *Enterobacteriaceae*, while *Pseudomonas aeruginosa* (PA) (n = 30) and *Acinetobacter baumannii* (AB) (n = 23) were the most common GNF GNB; only *Aeromonas* spp. (n = 9) were observed in the *Vibrionaceae*. Bacterial species and numbers of CR-GNB and CS-GNB were described in **Table 1**. There were 77 of 244 GNB isolates (31.6%) resistant to carbapenems, and the rate of carbapenem resistance appeared to be increasing year on year, from 20.4% in 2015 to 42.6% in 2019 (*p* = 0.004) (**Figure 2A**). In the CR-GNB cohort, *Klebsiella pneumoniae* (n = 30; 39.0%) was the most frequently encountered strain, followed by *Acinetobacter baumannii* (n = 21; 27.3%) and *Escherichia coli* (n = 13; 16.9%) (**Table 1**).

**Table 1:** Distribution of bacterial species and numbers of CR-GNB and CS- GNB.

Bacterial species	CR-GN B(n=77,%)	CS-GNB (n=167,%)	Totals (n=244,%)
**Enterobacteriaceae**	45 (58.4)	128 (76.6)	173 (70.9)
Escherichia coli	13 (16.9)	53 (31.7)	66 (27.0)
enterobacter cloacae	2 (2.6)	13 (7.8)	15 (6.1)
Klebsiella pneumoniae	30 (39.0)	49 (29.3)	79 (32.4)
Klebsiella oxytoca	0	3 (1.8)	3 (1.2)
Prpteus spp.	0	3 (1.8)	3 (1.2)
Serratia marcescens	0	3 (1.8)	3 (1.2)
others	0	4 (2.4)	4 (1.6)
**Glucose non-fermenting GNB**	30 (38.9)	32 (19.1)	62 (25.4)
Pseudomonas aeruginosa	0	30 (18.0)	30 (12.3)
Acinetobacter baumannii	21 (27.3)	2 (1.2)	23 (9.4)
Acinetobacter junii	1 (1.3)	0	1 (0.4)
Stenotrophomonas maltophilia	8 (10.4)	0	8 (3.3)
**Vibrionaceae**	2 (2.6)	7 (4.2)	9 (3.7)
Aeromonas spp.	2 (2.6)	7 (4.2)	9 (3.7)

CR-GNB, carbapenem-resistant gram-negative bacteria; CS-GNB, carbapenem-sensitive gram-negative bacteria.

### 3.2 Demographic and clinical characteristics of patients with GNB BSI

The demographic and clinical characteristics of these patients are summarized in **Table 2**. Among these 244 patients with GNB, 226 (92.6%) were recognized as hospital-acquired bacteremia, which included all patients (n = 77; 100%) with CR-GNB and the majority (n = 149; 89.2%) with CS-GNB. In the study, the median age of the patients was 44 years (IQR, 29.25-53) and the majority were male (n = 136; 55.7%). At the same time, we observed that AML, ALL, and lymphoma were the predominant underlying diseases (110 cases, 64 cases, and 30 cases, respectively), accounting for 45.1%, 26.2%, and 12.3% of the total number of cases.

**Table 2:** Clinica and demographic characteristics of hematological patients with bloodstream infection caused by gram-negative bacteria based on the carbapenem resistance of strains.

Variables	Total (n=244)	CR-GNB (n=77)	CS-GNB (n=167)	*P* values
Demographics				
Gender,male	136 (55.7)	50 (64.9)	86 (51.5)	0.050
Age,Years,median (IQR)	44 (29.25-53)	44 (29-52)	44 (30-53)	0.637
Underlying disease				0.947
Acute lymphatic leukemia	64 (26.2)	22 (28.6)	42 (25.1)	
Acute myeloid leukemia	110 (45.1)	33 (42.9)	77 (46.1)	
lymphoma	30 (12.3)	9 (11.7)	21 (12.6)	
Myelodysplastic syndrome	7 (2.9)	3 (3.9)	4 (2.4)	
Multiple Myeloma	6 (2.5)	1 (1.3)	5 (3.0)	
Aplastic anemia	16 (6.6)	6 (7.8)	10 (6.0)	
others	11 (4.5)	3 (3.9)	8 (4.8)	
incomplete remission status of underlying disease	178 (73.0)	62 (80.5)	116 (69.5)	0.071
Hypoproteinemia ^b^	100 (41.0)	47 (61.0)	53 (31.7)	<0.001
Comorbidities				
Diabetes mellitus	19 (7.8)	6 (7.8)	13 (7.8)	0.998
Hepatobiliary disease	28 (11.5)	7 (9.1)	21 (12.6)	0.427
Cardiovascular diseases	29 (11.9)	8 (10.4)	21 (12.6)	0.624
pulmonary infection at the time of BSI	121 (49.6)	47 (61.0)	74 (44.3)	0.015
Corticosteroid therapy before BSI ^a^	86 (35.2)	34 (44.2)	52 (31.1)	0.048
immunosuppressive therapy before BSI ^a^	18 (7.4)	5 (6.5)	13 (7.8)	0.720
Duration of neutropenia, Days,median (IQR)	5 (3-9.75)	8 (5-17)	4 (2-7)	<0.001
Duration of neutropenia ≥ 6 days ^a^	111 (45.5)	55 (71.4)	56 (33.5)	<0.001
Mucosal barrier damage ^b^	94 (38.5)	42 (54.5)	52 (31.1)	<0.001
Hospital-acquired BSI	226 (92.6)	77 (100)	149 (89.2)	0.003
Antifungal agents use within 30 days before BSI	137 (56.1)	62 (80.5)	75 (44.9)	<0.001
Antibiotics use within 30 days before BSI				
Carbapenems	96 (39.5)	52 (67.5)	44 (26.5)	<0.001
Aminoglycosides	23 (9.4)	11 (14.3)	12 (7.2)	0.078
Quinolones	41 (16.8)	15 (19.5)	26 (15.6)	0.448
Cephalosporin/β-lactamase inhibitor combinations	81 (33.2)	41 (53.2)	40 (24.0)	<0.001
Piperacillin-tazobactam	46 (18.9)	20 (26.0)	26 (15.6)	0.053
Tigecyclines	41 (16.8)	22 (28.6)	19 (11.4)	0.001
Indwelling central venous catheter ^a^				0.460
PICC	164 (67.2)	56 (72.7)	108 (64.7)	
PORT	34 (13.9)	9 (11.7)	25 (15.0)	
Indwelling urinary catheter ^a^	11 (4.5)	7 (9.1)	4 (2.4)	0.019
28-Day mortality (n=240)	68 (28.3)	40 (52.6)	28 (17.1)	<0.001
Inappropriate empirical therapy (n=243)	51 (21.4)	49 (63.6)	3 (1.8)	<0.001
Appropriate empirical therapy (n=243)	191 (78.6)	28 (36.4)	163 (98.2)	<0.001
Septic shock ^c^	53 (21.7)	27 (35.1)	26 (15.6)	0.001

a Before bloodstream infection within 30 days.

b At the time of bloodstream infection.

c Before the result of Antibiotic susceptibility testing.

PICC, peripherally inserted central catheter; PORT, implantable venous access port; BSI, bloodstream infection; CR-GNB, carbapenem-resistant gram-negative bacteria; CS-GNB, carbapenem-sensitive gram-negative bacteria; IQR, interquartile range.

### 3.3 Establishment and validation of the nomogram for early predicting the risk probability of carbapenem resistance in HM patients with GNB BSI

#### 3.3.1 Risk factors for carbapenem resistance in patients with GNB BSI

**Figure 3:**
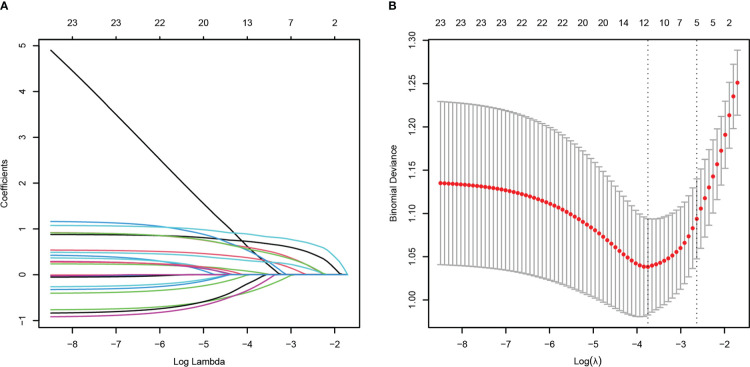
Potential risk factors selection for carbapenem resistance using the least absolute shrinkage and selection operator (LASSO) regression model with 10-fold cross validation. LASSO coefficient profiles of 23 variables **(A)**. A coefficient profile plot was constructed from the log (lambda) sequence **(B)**. The number of characteristic variables was filtered by drawing dotted vertical lines at lambda.min (left dotted line) and lambda.1se (right dotted line) respectively according to the minimum criterion. LASSO, least absolute shrinkage and selection operator; SE, standard error.

To minimize the risk of multicollinearity of variables in the prediction model of this study, LASSO regression analysis was performed to filter potential risk factors for carbapenem resistance among hematological patients with GNB BSI. **Figure 3** illustrated the selection process of potential risk factors for carbapenem resistance using the LASSO regression model. As demonstrated in **Figure 3B**, lambda.lse (λ = 0.07182097) (right dotted line) was identified as the optimal lambda for five variables with non-zero coefficients. These variables with potential risk of carbapenem resistance included duration of neutropenia ≥ 6 days before BSI, previous exposure to cephalosporin/β-lactamase inhibitor combinations, previous exposure to carbapenems, antifungal agents use within 30 days before BSI, and hypoproteinemia. To further analyze independent risk factors for CR-GNB BSI, five variables selected from the LASSO regression model were incorporated into the multivariate logistic regression model. Ultimately, multivariate logistic analysis (**Table 3** Model-A) demonstrated that duration of neutropenia ≥ 6 days before BSI (OR 2.764, 95% CI: 1.437-5.317; *p* = 0.002), hypoproteinemia (OR 2.249, 95% CI: 1.196-4.227; *p* = 0.012), previous exposure to carbapenems (OR 3.460, 95% CI: 1.822-6.571; *p* < 0.001) and previous exposure to cephalosporin/*β*-lactamase inhibitors (OR 2.162, 95% CI: 1.134-4.120; *p* = 0.019) were independent risk factors of CR-GNB BSI.

**Table 3:** Multivariate analysis of risk Factors for carbapenem resistance (Model A) and 28-day all-cause mortality (Model B) in patients with hematological diseases suffering from bloodstream infections caused by gram-negative bacteria.

Variables	*B*	*P*	*OR*	*95%CI*
Model(A)				
Duration of neutropenia ≥ 6 days ^a^	1.017	0.002	2.764	(1.437-5.317)
Hypoproteinemia ^b^	0.810	0.012	2.249	(1.196-4.227)
Previous exposure to carbapenems ^a^	1.241	<0.001	3.460	(1.822-6.571)
Previous exposure to Cephalosporin/β-lactamase inhibitor combinations ^a^	0.771	0.019	2.162	(1.134-4.120)
Model(B)				
Hypoproteinemia ^b^	0.783	0.049	2.189	(1.002-4.781)
Incomplete remission status of underlying disease ^b^	2.290	0.001	9.880	(2.569-37.989)
Isolation of CR-GNB	1.459	<0.001	4.304	(1.921-9.644)
Septic shock ^c^	2.830	<0.001	16.950	(6.846-41.967)

a Before bloodstream infection within 30 days.

b At the time of bloodstream infection.

c Before the result of Antibiotic susceptibility testing.

CR-GNB, carbapenem-resistant gram-negative bacteri.

#### 3.3.2 Establishment and validation of the nomogram

**Figure 4:**
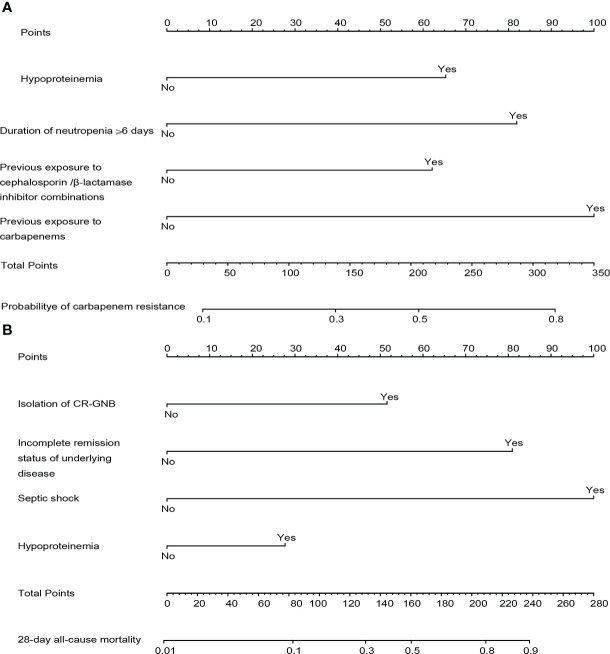
The constructed nomograms for predicting risk probability of carbapenem resistance and 28-day all-cause mortality in gram-negative bacteremia among patients with hematological diseases, abbreviated as the carbapenem resistance nomogram **(A)** and the prognosis nomogram **(B)**. Each predictor has corresponding points, and the total score for an individual patient could be obtained by summing up all scores. CR-GNB, carbapenem-resistant gram-negative bacteria.

Combining clinical knowledge and the results of previous studies (Lalaoui et al., 2020), we finally constructed a nomogram containing four variables (hypoproteinemia, duration of neutropenia ≥ 6 days, previous exposure to carbapenems, and previous exposure to cephalosporins/*β*-lactamase inhibitors, respectively) to personalize the prediction of the probability of carbapenem resistance in HM patients with GNB BSI, which was abbreviated as the carbapenem resistance nomogram (**Figure 4A**). In the nomogram, each variable was represented visually with a corresponding score. The total points of a patient with GNB BSI could be calculated by adding the scores of each predictive variable, which was corresponded to the predicted probability of carbapenem resistance.

**Figure 5:**
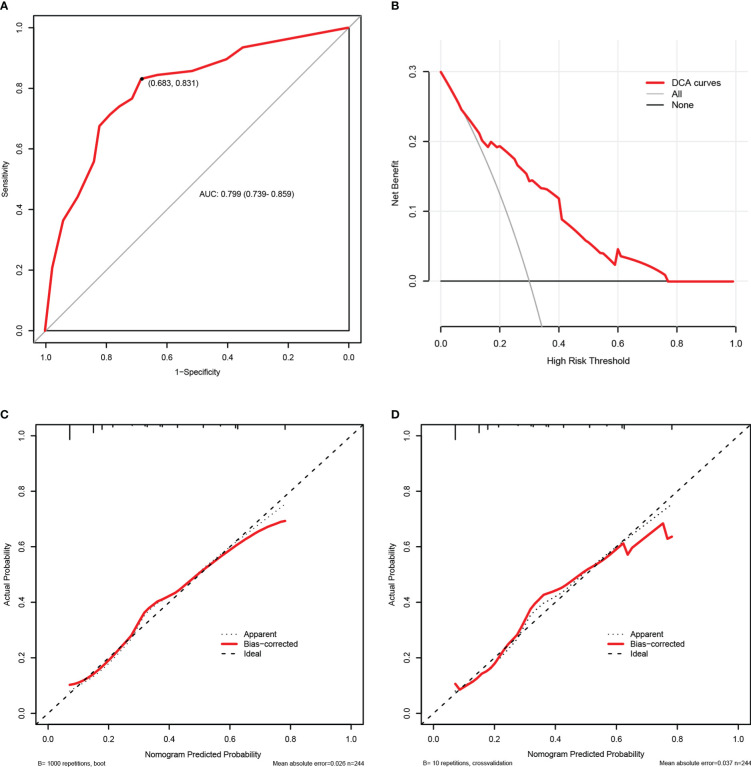
The internal validation of the carbapenem resistance nomogram according to bootstrap method and 10-fold cross validation. Receiver operating characteristic (ROC) curves of the carbapenem resistance nomogram **(A)**. Decision curve analysis (DCA) of the carbapenem resistance nomogram **(B)**. Calibration plots of the carbapenem resistance nomogram based on bootstrap method **(C)** and 10-fold cross validation **(D)**.

The internal validation results of this nomogram were depicted graphically in **Figure 5**. As shown in **Figure 5A**, the AUC values of the carbapenem resistance nomogram was 0.799 (0.739-0.859), and the sensitivity and specificity were 68.3% and 83.1%, respectively. Subsequently, overfitting or underfitting was assessed by bootstrapping and 10-fold cross validation method, and the model was found to be well-fitting. The modified C-index for the nomogram were 0.788 and 0.781 according to bootstrapping and 10-fold cross validation, respectively, indicating that the predictive model possessed a favorable capacity for discrimination of carbapenem resiatance. In addition, the accuracy or C-index of this model based on other cross validation was summarized in **Table SII**. Similarly, it was worth noting that the calibration plots of the prediction model were all extremely approximated to the corresponding actual observed curves for both the bootstrapping and 10-fold cross validation (**Figures 5C, D**), suggesting that the nomogram provided a satisfactory prediction accuracy. Meanwhile, we also assessed the clinical applicability of the prediction model using DCA, as displayed in **Figure 5B**, and found that the model had excellent clinical utility.

### 3.4 Treatments and outcomes

To evaluate the risk factors for 28-day mortality following the onset of GNB BSI, we eliminated cases with unavailable follow-up data (4 cases). Eventually, a total of 240 episodes of GNB BSI were analyzed. Following the collection of blood culture specimens, an empirical antimicrobial regimen was implemented for all febrile patients immediately in accordance with Clinical Practice Guidelines for the management of patients with febrile neutropenia in the USA and Europe, with 77.9% (187/240) of patients receiving appropriate empirical therapy. The 28-day all-cause mortality in HM patients with GNB BSI was 28.3% (68/240) and 44.1% (30/68) of non-survivors suffered from insufficient empirical antimicrobial therapy (**Table SI**). Moreover, there was a significantly higher mortality in CR-GNB BSI patients than that in CS-GNB BSI patients (40/77, 52.6% vs 28/167, 17.1%; *P* < 0.001). Meanwhile, in our cohorts, a considerably greater proportion of the CR-GNB patients received inappropriate empirical treatment than CS-GNB patients (48/76, 63.2% vs 3/155, 1.9%; *P* < 0.001). A Kaplan-Meier survival analysis also demonstrated the higher possibility of mortality among patients infected with CR strains (*P* < 0.001) (**Figure 2B**).

### 3.5 Establishment and validation of the nomogram for early predicting the risk probability of 28-day mortality in HM patients with GNB BSI

#### 3.5.1 Risk factors for 28-day all-cause mortality in patients with GNB BSI

**Figure 6:**
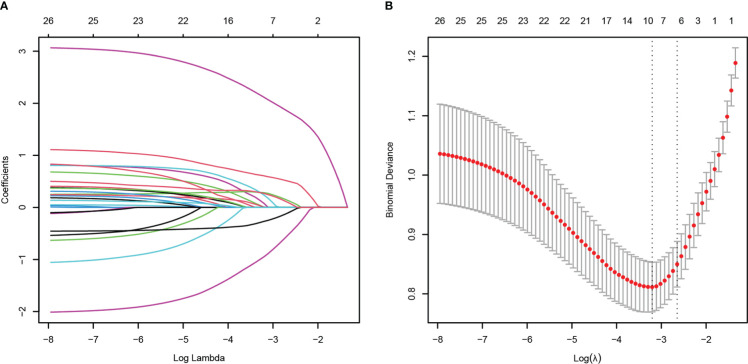
Potential risk factors selection for 28-day all-cause mortality using the least absolute shrinkage and selection operator (LASSO) regression model with 10-fold cross validation. LASSO coefficient profiles of 26 variables **(A)**. A coefficient profile plot was constructed from the log (lambda) sequence **(B)**. The number of characteristic variables was filtered by drawing dotted vertical lines at lambda.min (left dotted line) and lambda.1se (right dotted line) respectively according to the minimum criterion. LASSO, least absolute shrinkage and selection operator; SE, standard error.

The HM patients with GNB BSI were classified into survivor and non-survivor groups according to the clinical outcomes of the 28th day. As with the approach for screening the most appropriate variables of carbapenem resistance, we identified possible prognostic predictors through lasso regression and multivariate logistic regression analysis. As presented in **Figure 6**, isolation of CR-GNB, hypoproteinemia, previous exposure to carbapenems, incomplete remission status of underlying disease, septic shock, and appropriate empirical therapy were potential variables screened to predict clinical prognosis with the LASSO regression analysis. Multivariate logistic regression analysis (**Table 3** Model-B) further indicated that the following factors were independently associated with a higher risk of mortality for GNB BSI: hypoproteinemia (OR 2.189, 95%CI: 1.002-4.781; *P =* 0.049), septic shock (OR 16.950, 95%CI: 6.846-41.967; *P* < 0.001), incomplete remission status of underlying diseases (OR 9.880, 95%CI: 2.569-37.989; *P* = 0.010), and isolation of CR-GNB (OR 4.304, 95%CI: 1.921-9.644; *P* < 0.001).

#### 3.5.2 Establishment and validation of the nomogram

The four independent prognostic predictors (hypoproteinemia, septic shock, incomplete remission status of underlying diseases, and isolation of CR-GNB) were incorporated to generate a predictive nomogram for early individualized prediction of 28-day all-cause mortality in patients with GNB BSI, which was abbreviated as the prognosis nomogram (**Figure 4B**).

**Figure 7:**
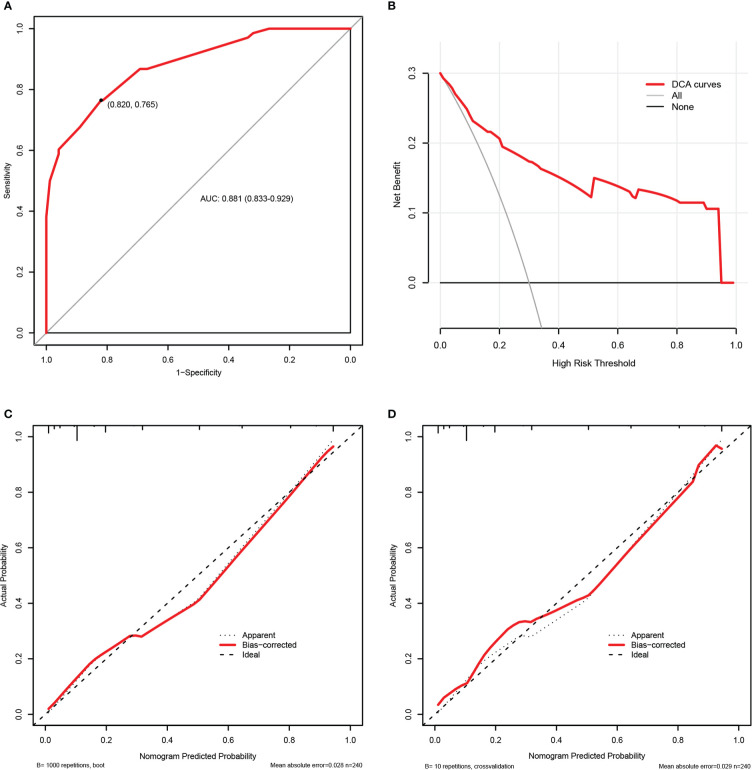
The internal validation of the prognosis nomogram according to bootstrap method and 10-fold cross validation. Receiver operating characteristic (ROC) curves of the prognosis nomogram **(A)**. Decision curve analysis (DCA) of the prognosis nomogram **(B)**. Calibration plots of the prognosis nomogram based on bootstrap method **(C)** and 10-fold cross validation **(D)**.

**Figure 7** exhibited the performance evaluation findings of the nomogram using bootstrapping and 10-fold cross validation, respectively. According to **Figure 7A**, the AUC of the prognosis nomogram was 0.881 (0.833-0.929), with a sensitivity of 82.0% and a specificity of 76.5%. When validated internally by bootstrapping and 10-fold cross validation method, the modified C-indexes of the nomogram were 0.873 and 0.887, respectively, revealing that the predictive model had a superior discriminative ability for the identification of poorer clinical prognosis. In addition, **Table SII** summarized the accuracy or C-index of this model based on additional cross validation. The calibration curves for both the bootstrapping and 10-fold cross validation demonstrated a relatively high agreement between prediction and actual observation (**Figures 7C, D**). Likewise, the decision curve analysis (DCA) demonstrated that the prognostic nomogram model had greater net benefits for identifying clinical outcomes with worse prognoses (**Figure 7B**).

## 4 Discussion

The clinical management of bloodstream infection (BSI) is crucial to the overall prognosis of patients with hematological disorders. In the present research, we systematically summarized the clinical characteristics of Gram-negative bacteremia in patients with hematological diseases at our hospital and screened the potential high risk factors of 28-day all-cause mortality and carbapenem resistance using LASSO regression analysis and multivariate logistic regression analysis. On the basis of the aforementioned predictive indicators, we established two nomograms to earlier identify the probability of carbapenem resistance at the time of the isolation of GNB and the 28-day mortality risk for each patient encountering Gram-negative bacteremia in the hematology department. Our nomograms exhibited relatively excellent performance in terms of discrimination, calibration, and clinical practicality, as measured by the C-index or AUC values, calibration plots, and DCA curves. Therefore, they might be straightforward and practical pictorial prediction tools that provide beneficial information for clinicians in the prevention and control of BSI. This appears to be, to our knowledge, a first report focusing on the development and validation of nomograms for early personalized prediction of the possibility of carbapenem resistance and mortality risk in patients with hematological disorders suffering from BSI caused by GNB.

In accordance with earlier research findings (Trecarichi and Tumbarello, 2014; Di Domenico et al., 2021), GNB remained the most prevalent microorganism species (377/679, 55.5%) causing BSI in our cohort of patients by far (**Figure 1**). However, the variety of pathogenic microorganisms varies according to geographic areas, years, and other factors. Our data indicated that KP was the predominantly isolated pathogen, followed by EC, and PA during the study period (2015-2019). These data differed slightly from what has been reported in association with epidemiological studies performed on HM patients in other countries and in other cities of our country, in which EC represented the most frequently isolated bacteria, followed by PA or KP (Trecarichi and Tumbarello, 2014; Andria et al., 2015; Chen et al., 2017; Islas-Muñoz et al., 2018; Di Domenico et al., 2021). Previous researches have shown that carbapenem resistance in GNB has become a significant problem worldwide and a new public health threat in view of limited antimicrobial treatment options and high mortality (Righi et al., 2017; Nordmann and Poirel, 2019). Notably, the overall trend of carbapenem resistance in our research has increased from 20.4% in 2015 to 42.6% in 2019, which is consistent with the finding of the national bloodstream infection surveillance program conducted in China from 2014 to 2019 (Chen et al., 2022). In agreement with previous published studies conducted in HM patients (Andria et al., 2015; Trecarichi et al., 2016; Lalaoui et al., 2020), the overall mortality of CR-GNB BSI patients was significantly higher than that of CS-GNB BSI patients in our study(52.6% vs 17.1%; P < 0.001), and isolation of CR-GNB was independently correlated with 28-day all-cause mortality. These results highlight the necessity of regular surveillance to comprehend the clinical epidemiological characteristics of BSI among hematological patients in the local hospital.

In this study, the 28-day all-cause mortality of GNB BSI was 28.3%, and the majority of non-survivors (58.8%) suffered from BSI caused by CR-GNB (**Table SI**). Until now, a variety of risk factors have been demonstrated to be associated with carbapenem resistance in gram-negative bacteria, of which exposure to carbapenems before infections represented the most frequently observed risk factor across the different pathogens and types of infection (Satlin et al., 2016; Righi et al., 2017; Ting et al., 2018; Xiao et al., 2020; Zhao et al., 2020; Palacios-Baena et al., 2021). The use of carbapenems prior to BSI was also recognized as an independent risk factor for CR-GNB BSI in our analysis. Furthermore, a multi-center study conducted in China concerning the correlation between antibiotics consumption and carbapenem resistance equally revealed that carbapenem resistance in GNB was positively and significantly correlated with increased consumption of carbapenem antibiotics (Yang et al., 2018). Such a correlation may be attributed to the high selective pressure of carbapenems exposure. Notably, Laurence et al (Armand-Lefevre et al., 2013)found that even short-duration exposure to carbapenems may increase the risk of colonization or infection with carbapenem-resistant pathogens. Our study also suggested that previous use of cephalosporins/*β*-lactamase inhibitor combinations was associated with CR-GNB BSI. As with our research findings, there was an analogous positive correlation between prior cephalosporins/*β*-lactamase inhibitor combinations usage and carbapenem resistance in several other studies (Satlin et al., 2016; Liang et al., 2021). A plausible interpretation for this may be that broad-spectrum antibiotics have the potential to cause alteration of intestinal flora and may select for and promote the growth of carbapenem-resistant organisms in intestinal microbiota. Therefore, antimicrobial stewardship programs are essential to alleviate the emergence of resistance owing to the selective pressure of antibacterial usage. Zou et al (Zou et al., 2014)indicated that reduced use of antimicrobials was parallel with the amelioration of bacterial resistance without deteriorating medical quality indicators, which underlines the importance and feasibility of antimicrobial stewardship.

Previous retrospective clinical studies (Murthy et al., 2018; Omiya et al., 2021) found that, in addition to previous antibacterial usage, neutropenia was an another significant independent risk factor for carbapenem resistance. However, the relationship between the duration of neutropenia and carbapenem resistance has received limited consideration. Our study found that the duration of neutropenia ≥ 6 days was associated with a higher risk of carbapenem resistance in HM patients with BSI. Meanwhile, hypoproteinemia was a statistically screened independent risk factor associated with carbapenem resistance in our study, and yet has rarely been observed to be related to carbapenem resistance in previous studies. Given that hypoproteinemia has been linked to disease progression and clinical prognosis in different clinical settings (Murthy et al., 2018; Aziz et al., 2020; Omiya et al., 2021; Jiang et al., 2022), it was also incorporated into the plotting of the carbapenem resistance nomogram.

According to our findings, the isolation of CR-GNB, hypoproteinemia, the incomplete remission status of underlying diseases, and septic shock were strongly associated with a high risk of clinical worsening prognosis. Carbapenem-resistant GNB tended to be multi-drug resistant or even pan-resistant and had a limited choice of available antibiotic types. Trecarichi et al. (2016) and Andria et al., (2015) proved that inappropriate empirical antimicrobial regimens were strongly associated with high mortality and the majority of patients with CR-GNB BSI failed to obtain appropriate empirical antibiotic treatment. In our study, 44.1% (30/68) of non-survivors underwent inadequate empirical antimicrobial therapy, with a significantly higher proportion of inappropriate empirical therapy in the CR-GNB group than that in the CS-GNB group (63.6% vs 1.8%; P < 0.001). At the same time, we found that 15.6% of patients with BSI in the CS-GNB group progressed to sepsis shock and even clinical death. This could not be ruled out in connection with the increasing emergence of strains with high virulence and pathogenicity, which are active against most antibiotics *in vitro* but possess a high mortality risk (Zhang et al., 2016; Russo and Marr, 2019). It has been documented that infections caused by highly virulent Klebsiella pneumoniae (hvKP) most frequently occurred in the Asia-Pacific region (Russo and Marr, 2019). Additionally, a systematic multi-center study of hvKP infection performed in China reported that the percentage of hvKP infection varied from 8.33% to 73.9%, with the highest prevalence in Wuhan (Zhang et al., 2016). Of course, it is essential to carry out more studies in the future to confirm the presence of highly virulent GNB in our hematology.

In addition, we also found that septic shock was the predominant and significant predictor for 28-day mortality in our nomogram. Not surprisingly, septic shock, as a clinical manifestation of the severity of infections and organ dysfunction, has been detected to be associated with high mortality in several previous studies involving patients with HM and the general populations (Andria et al., 2015; Chumbita et al., 2022; Xiao et al., 2020; Zhang et al., 2020). In the current study, we could understand that 60.3% (41/68) of non-survivors underwent septic shock, of which 50.9% (27/53) of patients were categorized as CR-GNB BSI. Therefore, in clinical practice, the severity of the infection plays a crucial role in the prognosis. As demonstrated by Chumbita et al. (2022), when administered with inappropriate experimental antibiotic therapy, septic shock was associated with extremely high mortality in febrile neutropenic patients with BSI. Apparently, this conclusion further highlights the importance of epidemiological surveillance in different hospitals. Furthermore, the stage of hematological disease was a non-negligible variable in our study as well, where 95.6% (65/68) of non-survivors failed to attain complete remission status. It might be that when patients were in the status of incomplete remission, the tumor load was high and a more effective high-dose chemotherapy regimen was required.

In recent years, nomograms have become increasingly important in the clinical management of infectious diseases (Dong et al., 2021; He et al., 2022), while are rarely implemented in patients with hematological diseases. In hematological patients with BSI, the majority of studies have focused on the description of outcomes for regression models or the development of predictive scoring systems (Satlin et al., 2016; Xiao et al., 2020), which are not as simple and intuitive as nomograms. In this study, we improved the accuracy of predictor selection using LASSO analysis and built quantitative nomograms to provide a quick and easy evaluation of the probability of carbapenem resistance and mortality risk. In accordance with the TRIPOD statement (Collins et al., 2015), our nomograms were internally validated using the original study sample based on bootstrapping and 10-fold cross validation method, and exhibited relatively good predictive performance. Because of this, the findings emphasize the importance of antibiotic stewardship and the identification of patients at high risk of carbapenem resistance.

However, we have to acknowledge the existence of several shortcomings in the study. First, this is a single-center retrospective study, and the lack of an independent external validation cohort is a drawback of nomogram models, despite the relatively excellent predictive performance of nomogram models demonstrated with internal validation based on both the bootstrap method and 10-fold cross validation. Furthermore, other variables in this study that could not be identified from the electronic medical record system, such as the intestinal colonization of carbapenem-resistant organisms and the source of BSI, which are relatively associated with the development of carbapenem resistance and the clinical prognosis separately, may interfere with the ultimate results of predictive models. Thus, a more comprehensive multi-center prospective study in the future is required to demonstrate the reproducibility of nomograms and further revise our models, which also indicates the necessity of cyclic evaluations for similar clinical events. Secondly, the detection performance of microbiological methods and the use of antimicrobials prior to specimen collection may underestimate the amount of microbial growth in clinical cultures and influence the final results to some extent. In spite of these limitations, our study still provides a certain reference value to adjust management strategies for the prevention and control of Gram-negative bacteremia in patients with hematological diseases.

In summary, Gram-negative bacteria remain the leading pathogens of bloodstream infections in the department of hematology. Carbapenem resistance is associated with a worse prognosis in patients with GNB BSI. Meanwhile, individualized risk prediction models were established and validated in this study for predicting the risk of 28-day all-cause mortality and carbapenem resistance in gram-negative bacteremia among patients with hematological diseases. These models could serve as simple, reliable tools to make individualized risk predictions of clinical events and to provide useful information for clinicians in the management of infection control measures.

## Data availability statement

The raw data supporting the conclusions of this article will be made available by the authors, without undue reservation.

## Ethics statement

The studies involving human participants were reviewed and approved by the Ethics Committee of Union Hospital, Tongji Medical College, Huazhong University of Science and Technology. Written informed consent from the participants’ legal guardian/next of kin was not required to participate in this study in accordance with the national legislation and the institutional requirements.

## Author contributions

JH and HW designed the study. XJ, SD, XZ, ZX, KW, and XD collected data. XJ and SD analyzed the data and wrote the article. JH and HW critically reviewed the manuscript. All authors contributed to the article and approved the submitted version.
