# Supplementary material for: Development and validation of nomograms for predicting the risk probability of carbapenem resistance and 28-day all-cause mortality in gram-negative bacteremia among patients with hematological diseases
Source: Front Cell Infect Microbiol. 2023 Jan 5;12:969117. doi: 10.3389/fcimb.2022.969117 (PMC9849754; doi:10.3389/fcimb.2022.969117)
Supplement: Supplementary file 1 [file Table_1.pdf]

Supplementary materials

Table SI Univariate analysis of risk factors for 28-day all-cause mortality in 240 hematological patients with gram-negative bacteria bloodstream infection

**Variables**	**Total (n=240)**	**Non-survival （n=68）**	**Survival （n=172）**	***P* values**
Demographics				
Gender,male	134 (55.8)	42 (61.8)	92 (53.5)	0.245
Age,Years,median (IQR)	44 (30-53)	46 (37-54)	41.5 (28-53)	0.071
Age ≥ 40 years	142 (59.2)	49 (72.1）	93 (54.1)	0.011
Underlying disease				0.176
Acute lymphatic leukemia	62 (25.8)	18 (26.5)	44 (25.6)	
Acute myeloid leukemia	109 (45.4)	26 (38.2)	83 (48.4)	
lymphoma	30 (12.5)	11 (16.2)	19 (11.0)	
Myelodysplastic syndrome	7 (2.9)	5 (7.4)	2（1.2)	
Multiple Myeloma	6 (2.5)	1 (1.5)	5 (2.9)	
Aplastic anemia	15 (6.3)	4 (5.9)	11 (6.4)	
others	11 (4.6)	3（4.4)	8 (4.7)	
Comorbidities				
Diabetes mellitus	19 (7.9)	9 (13.2)	10 (5.8)	0.055
Hepatobiliary disease	28 (11.7)	11 (16.2)	17 (9.9)	0.171
Cardiovascular diseases	28 (11.7)	9 (13.2)	19 (11.0)	0.634
pulmonary infection at the time of BSI	118 (49.2)	43 (63.2)	75 (43.6)	0.006
Corticosteroid therapy before BSI	85 (35.4)	31 (45.6)	54 (31.4)	0.038
Immunosuppressive therapy before BSI	17 (7.1)	3 (4.4)	14 (8.1)	0.310
Damage to mucosal barrier [a]	93 (38.8)	33 (48.5)	60 (34.9)	0.051
Duration of neutropenia	5 (3-9)	8 (4-15)	4 (2-7.5)	0.022
Duration of neutropenia ≥ 7 days [a]	90 (37.5)	39 (57.4)	51 (29.7)	<0.001
Antifungal agents use within 30 days before BSI	136 (56.7)	51 (75.0)	85 (49.4)	<0.001
Antibiotics use within 30 days before BSI				
Carbapenems	95 (39.6)	43 (63.2)	52 (30.2)	<0.001
Aminoglycosides	23 (9.6)	8 (11.8)	15 (8.7)	0.470
Quinolones	40 (16.7)	17 (25.0)	23 (13.4)	0.029
Cephalosporin/β-lactamase	80 (33.3)	31 (45.6)	49 (28.5)	0.011

inhibitor combinations				
Piperacillin-tazobactam	45 (18.8)	14 (20.6)	31 (18.0)	0.646
Tigecyclines	40 (16.7）	20 (29.4)	20 (11.6)	0.001
incomplete remission status of underlying disease [b]	174 (72.5)	65 (95.6）	109 (63.4）	<0.001
Hypoproteinemia [b]	99 (41.3)	46 (67.6)	53 (30.8)	<0.001
Hospital-acquired BSI	224 (93.3)	64 (94.1)	160 (93.6)	0.759
Indwelling central venous catheter [b]				0.574
PICC	163 (67.9)	44 (64.7)	119 (69.2)	
PORT	34 (14.2)	9 (13.2)	25 (14.5)	
Indwelling urinary catheter [b]	11 (4.6)	7 (10.3)	4 (2.3)	0.008
Septic shock [c]	53 (22.1)	41 (60.3)	12 (7.0)	<0.001
Acute respiratory failure	47 (19.6)	39 (57.4)	8 (4.7)	<0.001
Acute heart failure	19 (7.9)	14 (20.6)	5 (2.9)	<0.001
Altered state of consciousness	10 (4.2)	9 (13.2)	1 (0.6)	<0.001
Inappropriate empirical therapy	53 (22.1)	30 (44.1)	23 (13.4)	<0.001
appropriate empirical therapy	187 (77.9)	38 (55.9)	149 (86.6)	<0.001
Isolation of CR-GNB	76 (31.7)	40 (58.8)	36 (20.9)	<0.001

[a] Before bloodstream infection within 30 days

[b] At the time of bloodstream infection

[C] Before the result of Antibiotic susceptibility testing

Abbreviations: PICC, peripherally inserted central catheter; PORT, implantable venous access port; BSI, bloodstream infection; CR-GNB, carbapenem-resistant gram-negative bacteria; CS-GNB, carbapenem-sensitive gram-negative bacteria; IQR, interquartile range

Table SII The performance evaluation of the carbapenem resistance nomogram (Model A) and the progonosis nomogram (Model B) based on other cross validation methods

Indicators	Hold-out cross validation (7:3)	10-fold cross validation	Leave-one-out cross validation	Bootstrapping with 10-fold cross validation
**Nomogram (A)**				
C-index	0.679	0.781	0.760	0.786
Accuracy	0.712	0.739	0.725	0.739
Kappa	0.341	0.356	0.334	0.368
**Nomogram (B)**				
C-index	0.947	0.888	0.847	0.877
Accuracy	0.887	0.821	0.808	0.829
Kappa	0.722	0.539	0.510	0.554

Annotation: the C-index or accuracy was used to evaluate the discrimination of a predictive model. The C-index > 0.7 are considered to have relatively good discriminative accuracy. the kappa value was used to measure the stability of a predictive mode. kappa < 0.2: slight consistency; kappa between 0.2 and 0.4: fair consistency; kappa between 0.4 and 0.6: moderate consistency; kappa between 0.6 and 0.8: substantial consistency; kappa > 0.8: almost perfect.
